# Current policies and practices for the provision of diabetes care and self-management support programmes for older South Africans

**DOI:** 10.4102/phcfm.v11i1.2053

**Published:** 2019-08-29

**Authors:** Mahmoud Werfalli, Katherine Murphy, Sebastiana Kalula, Naomi Levitt

**Affiliations:** 1 Chronic Disease Initiative for Africa, Cape Town; and Department of Medicine, Division of Endocrinology and Diabetic Medicine, University of Cape Town, Observatory, Cape Town, South Africa; 2 The Albertina and Walter Sisulu Institute of Ageing in Africa, Cape Town; and Division of Geriatric Medicine, Department of Medicine, University of Cape Town, Cape Town, South Africa

**Keywords:** older patients with diabetes, diabetes care, diabetes self-management programmes, primary health care, South Africa

## Abstract

**Background:**

One of the most important primary health challenges currently affecting older people in South Africa (SA) is the increasing prevalence of non-communicable disease (NCD). Research is needed to investigate the current state of care and self-management support available to older diabetic patients in SA and the potential for interventions promoting self-management and community involvement.

**Aim:**

This study aimed to review current policies, programmes and any other interventions as they relate to older people with diabetes with a view to assess the potential for the development of a self-management programme for older persons attending public sector primary health care services in Cape Town, South Africa.

**Setting:**

Eighteen community health centres (CHCs) formed the sampling frame for the study.

**Methods:**

This study aimed to review current policies and programmes as they relate to older people with diabetes. It involved a documentary review and qualitative individual interviews with key informants in the health services and Department of Health.

**Results:**

Several national initiatives have sought to advance the health of older people, but they have only been partially successful. There are however multiple efforts to re-orientate the health-care system to focus more effectively on NCDs, which benefit older patients with diabetes. The establishment of community-based services to provide self-management support, promote health and ease access to medicine helps overcome many of the commonly cited barriers to care experienced by older patients. What may be equally important is that practitioners gain the communication skills and educational resources to effectively educate and counsel patients on lifestyle behaviour change and self-care management.

**Conclusion:**

This article alerts policy-makers and clinicians to some of the specific issues considered to be pertinent and important in the care and management of older diabetic patients. Many of these would also be applicable to older patients with other chronic conditions.

## Background

South Africa is undergoing the epidemiological transition typical of many low-to-middle income countries. Rapid urbanisation, attendant changes in diets and levels of physical activity along with population ageing have resulted in a significant increase in the burden of non-communicable disease (NCD), alongside a high prevalence of HIV, and violence and injuries, maternal and child mortality.^1^

Diabetes is associated with high levels of morbidity, multiple therapies and functional deterioration and as such poses challenges to health-care systems, individual patients and their families.^1^ Older persons are defined as individuals who are 60 years and older.^2^ For the older person, the complexity of managing diabetes is often exacerbated by increased frailty, functional limitation, changes in mental health and increased dependence.^1^ Social issues such as loss of independence, removal from the home environment and institutionalisation also commonly affect the quality of life and well-being of older people with diabetes and their ability to self-manage the condition. ^3^ Access to care is also often a challenge for older people, because of a lack of or the cost of transport, particularly in rural areas, as well as increasing physical disability and child caring responsibilities. Overcrowding and long waiting times at health facilities, fragmented or siloed care for multiple morbidities and poor communication with health-care providers are further barriers to care.^4,5^ All these issues impact on the capacity of older diabetes patients for self-care and glycaemic control. They also contribute to poor health outcomes and the high costs of diabetes treatment and care. Interventions which address the unique needs of older people with diabetes may, therefore, be warranted and may help reduce diabetes-related morbidity and associated health-care costs.^6^

The only South African document which specifically addresses the unique needs of the older diabetic patient is the 2017 Society for Endocrinology, Metabolism and Diabetes of South Africa (SEMDSA) guideline.^7^ According to the guideline, the comprehensive goals of diabetes management in older individuals are not significantly different from those of their younger counterparts, with glycaemic control and the reduction of other risk factors for macrovascular and microvascular disease remaining the key factors for optimal management. Lifestyle modification is still advised, and if there are no contraindications, physical activity should be an integral part of the treatment plan. The South African guideline is in line with the treatment recommendations in the Position Statement on behalf of the International Association of Gerontology and Geriatrics, the European Diabetes Working Party for Older People and the International Task Force of Experts of Diabetes.^8^ It recommends regular comprehensive geriatric assessments to identify related functional loss and the impact of disability, as well as regular screening for mood disorder and nutritional assessments.

Research is needed to investigate the current state of care and self-management support available to older diabetic patients in SA, the degree to which their needs are being met and the potential for interventions promoting self-management and community involvement in line with the WHO Innovative Care for Chronic Conditions (ICCC) Framework^10^ This study aimed to review current policies, programmes and any other interventions as they relate to older people with diabetes with a view to assess the potential for the development of a self-management programme for older persons attending public sector primary health-care services in Cape Town, SA.

## Methods

This study followed a qualitative, exploratory, descriptive design, using the methods of documentary review and individual interviews with key informants.

### Documentary review

A search was conducted with the aim of sourcing all documents that addressed the health needs of older people in SA and, more specifically, the needs of older people with diabetes. These included national and provincial government policy and planning documents, clinical guidelines for the treatment and management of diabetes, as well as commentaries or reviews of the SA health services compiled by civil society stakeholders or organisations. Relevant documents were sourced from SA government websites and internet searches using the keywords ‘older people in South Africa and health; SA policy on ageing; older persons and diabetes in SA/Western Cape’. Also, several documents were provided by the key informants, either during the interviews or subsequently by email.

### Key informant interviews

Qualitative, individual, face-to-face interviews were conducted with purposively selected key informants, who were chosen for their knowledge of the topic and their responsibility for implementation of national and/or provincial policies or programmes relating to diabetes in the public health sector, or for clinical practice in primary care clinics. Individuals were identified with the assistance of the Head of the Endocrinology Department at Groote Schuur Hospital in Cape Town. Interviews were conducted in a private room at the department and in three referring public sector primary health-care clinics (Retreat; Woodstock, Heideveld). An interview guide was developed to explore key informants’ knowledge of and views regarding diabetes self-management programmes for older persons attending public sector primary health-care services (Appendix 1). The interviews were about 30–45 min in duration and were conducted from December 2016 to April 2017. They were audio-taped and then transcribed verbatim.

## Data analysis

The method of qualitative content analysis was used to describe and interpret the data from the selected documents and the individual interviews.^9^ The analysis proceeded through the following steps: (1) reading through all the interview transcripts and documents to get a sense of the whole, (2) systematically identifying and coding relevant ‘meaning units’ and developing a coding list, (3) collating the coded data into categories and grouping these under higher order categories or more abstract headings, (4) the coherent integration of the analysis into a descriptive account of the mainly manifest content of the data and (5) the selection of compelling quotes to illustrate and summarise the findings in tables. In the initial stages, the authors M.W. and K.M. analysed the data independently and after that cooperated closely in developing an interpretive and descriptive account of the findings for the manuscript.

### Ethical considerations

The study was approved by the Human Ethics Committee of the University of Cape Town (HEC REF: 21/2013), and written consent was obtained from each participant interviewed.

## Findings

The findings below integrate information sourced through the documentary review and findings from the key informant interviews. They describe current policies, plans, guidelines and programmes which have relevance to the health needs and provision of care of older people with diabetes. Interviews were conducted with a total of five individuals: a senior official and policy-maker in the Department of National Health; a Director of Chronic Disease in the Western Cape Provincial Health Department; a family physician responsible for a primary care clinic in the Cape Town metro; a medical officer; and a health promoter. The researchers had approached other key informants, but unfortunately, they did not respond regardless of the email reminders that were sent to them weekly. The key to the labelling of these key informants is found in Table 1. The documents that were included in the documentary review are listed in Appendix 1, Table 1-A1.

**TABLE 1:** The key to the labelling of key informants.

Key informant	Position
KI 1	Director of Chronic Disease programme in the Western Cape Provincial Department of Health
KI 2	A senior official in the National Department of Health, responsible for NCD health policy and guidelines
KI 3	A family physician working in the primary care clinic in the Cape Town metro
KI 4	Operational manager of primary care clinic in the Cape Town metro
KI 5	Health Promoter in primary care clinic in the Cape Town metro

NCDs, non-communicable diseases.

## Theme 1: National initiatives for the advancement of the health and well-being of older persons in South Africa

An important milestone in advancing the interests of older people as a distinct group occurred in 2002 when SA signed the Declaration on Ageing at the Second World Assembly on Ageing in Madrid.^11^ This served as the basis for the 2002 SA Plan of Action of Ageing. The plan gives government the primary responsibility for implementation of the recommendations, but stressed that partnerships with civil society, academic institutions, the private sector and older persons themselves are critical to achieving its goals.^11^ In the section on health, the plan recommends that the National Department of Health (NDOH) review all health policies and strategies to ensure that services are more responsive to the specific needs of older people. There are specific recommendations that the NDOH should develop and implement programmes for the management of chronic health conditions that are more prevalent in old age and for the promotion of healthy and active lifestyles among older individuals to prevent illness and functional decline and in so doing generally improve quality of life.^12^

The next important national intervention was the passing of the *Older Person’s Act* in 2006, which provides for the protection of the elderly’s rights and the criminalisation of abuse. This was followed by programmes to educate the public on the rights of older persons in all provinces; the development of a national protocol on the management of abuse of older persons; the drawing up of minimum standards for residential care; and plans to promote the respect of older people at government service points (Operation Dignity).^13^ A parliamentary briefing by the Department of Social Development in 2013 provides further insight into the implementation of the Act and its challenges.^14^

According to Goodrick,^16^ these initiatives have only been partially successful in putting the health and welfare of older people higher on the national agenda and in improving the material and social well-being of the older South African population. This, he argues, is attributable primarily to policy prioritising the country’s young population, the focus on HIV and/or AIDS and an overall lack of awareness of the potential socio-economic and fiscal implications of population ageing. In his view, until ageing concerns are mainstreamed in policies, the development of services and facilities for the aged will remain relatively neglected. Further, a 2013 UN review of the Madrid International Plan of Action on Ageing concluded that there had been a decline in the well-being of older persons in Africa, including in SA since 2008 mainly because of demographic shifts, an increased burden of disease and inadequate government intervention.^16^

## Theme 2: National government action to address diabetes and other non-communicable diseases

The NDOH has drafted a Strategic Plan for the Prevention and Control of NCDs (2013), which is closely aligned to UN/WHO recommendations and places a strong emphasis on intervention in three distinct areas. Evidence suggested can produce rapid gains in reversing the NCD epidemic: (1) The prevention of NCDs and the promotion of health at a population, community and individual level to address the broader social determinants of health, (2) improved control of NCDs through the strengthening of the primary health system and (3) comprehensive monitoring of NCDs and their risk factors.^17^ At the population level, legislative and fiscal measures are in place to control tobacco, salt, trans-fats and sugar in beverages. Policy development on alcohol has, however, been slow mainly because of opposition from the industry. These interventions have had a positive impact, but more aggressive efforts are needed to ensure compliance.^18^ Several aspects of NCD surveillance need strengthening, notably the quality and completeness of information on mortality, the more regular monitoring of NCD risk factors and the quality of care.^19^

### Primary Health Care Re-engineering policy

As part of the NCD plan, the NDOH has introduced the Primary Health Care Re-engineering policy with the aim of strengthening the response to NCDs at this level in the health system. The policy envisages health facility-based chronic care teams working with ward-based outreach teams (WBOTS), comprising nurses and community health workers (CHWs) to deliver integrated NCD services, including follow-up and ongoing support to individuals in their households and communities. According to the SA Health Review (SAHR), while there has been an increase in the number of primary health-care facilities with functional chronic care teams and WBOTS, at this point, it is not clear how widely CHWs have been deployed and how well prepared they are for scaled-up community-based NCD prevention and management.^19^ Several studies in SA have demonstrated the capacity of CHWs to effectively execute specific NCD-related tasks, such as screening and health education, but they have also shown that there is a wide variation in their scope of practice and level of training, insufficient support and supervision and a lack of resources and supplies necessary for the performance of many of their tasks.^19,20^ Generally, patients still rely heavily on health facilities from NCD management and are still accessing health care at inappropriate levels.^20,21,22^. Quality improvement interventions in public health facilities are being implemented to meet the quality norms and standards to varying degrees. These include (1) the scaling up of the ideal clinic model, (2) infrastructure improvement across the health sector and (3) implementation of the WHO’s Workload Indicators for the Staffing Needs instrument. From a treatment perspective, the integrated chronic disease management (ICDM) model is a central part of the ideal clinic and re-engineering of primary care as a vehicle to improve the management of chronic conditions, including NCD.^23,24^

### Practical Approach to Care Kit

A further aspect of the re-engineering strategy is the implementation of the Practical Approach to Care Kit (PACK) in all primary care facilities. The Practical Approach to Care Kit is a programme comprising clinical guidance, an implementation strategy, health systems strengthening and monitoring and evaluation components. The Practical Approach to Care Kit Western Cape Adult started as a research project in the Eden district of the province and was subsequently launched as a provincial programme in March 2014.^21^ These are integrated, evidence-based clinical guidelines, which aim to improve the diagnosis and management of the most common conditions in primary care, including NCDs. A process evaluation in 2016 showed that there was widespread use of the guideline and that it was perceived as very useful by clinicians.^21^ KI 1 confirmed a positive response to PACK in the Western Cape and reported that it had been rolled out across the province. In the Western Cape province, the monitoring and evaluation of clinical and managerial performance in relation to chronic disease care is achieved through the Integrated Audit for Chronic Disease. The audit has been implemented across the province and is modelled on the Standard Treatment Guidelines, the Essential Medicines List and the PACK guideline. It includes the monitoring of five chronic conditions, namely diabetes, hypertension, asthma, Chroinc Obstructive Pulmonary Diseases (COPD) and epilepsy, and there is a section on patient satisfaction. By 2015, 187 primary health care facilities in the province were participating in the annual audit. According to KI 1, the audit has helped put systems, infrastructure and equipment in place to implement clinical guidelines and improve NCD outcomes:

‘From audit, I can see there is an improvement. Over the years, I have seen the effort that has been put in, and it is now paying off. It is slow, but it is paying off.’ (KI 1, Director, Western Cape Provincial Department of Health)

This opinion is borne out by an evaluation conducted by Essel et al.^27^ which showed that in districts where audits had been conducted for a period of time, there were marked improvements in clinical processes compared to districts that had only recently begun doing audits.^23^

### Advocating an integrated approach

Our key informant from the National Department of Health (KI 2) spent much of his interview explaining national efforts to promote a more integrated approach for chronic conditions:

‘We understand that there are many co-morbidities and we cannot continue to just treat diabetes or HIV for example, on its own. If there is specialised care for diabetes, for HIV and all the other common co-morbidities, how many different vertical and parallel services would we have? So, we are moving towards a much more integrated chronic care service.’ (KI 2, Senior Official, National Department of Health)

He did not just refer to medical services:

‘We would like this service to be as comprehensive as possible and include education and information and assisting with lifestyle change so that patients get more holistic care … Whilst there are specifics related to each disease, what is common (to the main chronic conditions) is that people need to take their medicine regularly, change their lifestyles and attend support groups. Self-management is extremely important because that means that patients rely on health workers less and come in less often, which give practitioners more time with individual patients.’ (KI 2, Senior Official, National Department of Health)

The provision of specific services for older diabetes patients was not supported by the key informants: ‘I don’t think we should set up different, parallel services for older people with diabetes. In our context, that is not a feasible option’ (KI 2, Senior Official, National Department of Health). It was, however, argued that the move towards integration of care would be of particular benefit to the older patient:

‘[*T*]he older one gets, the more chronic problems one is going to get and if you are going to be coming to see different practitioners on different days, your ability to control your health is going to get worse. You will need transport money each time and wait in more queues. That is going to put more stress on you and your family. So yes, the integrated model works extremely well for older people. I think it is a real bonus for them.’ (KI 2, Senior Official, National Department of Health)

### Community-based services

National policy fully endorses the role of non-government organisations (NGOs) in providing community-based health promotion and patient support:

‘Around the prevention and early detection of NCDs and the running of support groups, I have absolutely no doubt that the state needs to work hand in glove with NGOs.’ (KI 2, Senior Official, National Department of Health)

A further component of the national NCD policy is the Central Chronic Medicine Dispensing and Distribution (CCMDD) programme, which aims to more efficiently dispense medicines to chronic patients at external, convenient pick-up points, such as community venues and private pharmacies.^23^ Where this is working efficiently, the programme has been shown to reduce the need for stable HIV, hypertension and diabetes patients to visit public primary health-care clinics on a monthly basis to collect their medicines, with the positive effect of decongesting the clinics and reducing waiting times.^24^

In summing up the advantages of the integrated care policy (KI 2) said:

‘[*B*]y integrating the treatment of common chronic conditions and building self-assistance into our system by getting people to collect their medication and participate in self-management programmes, the fewer assessments patients will need. That should give the practitioner more quality time with each patient and more time for health promotion activities…that is the model we are aiming for.’ (KI 2, Senior Official, National Department of Health)

### Implementation of national policy

While the CCMDD programme has been initiated in most provinces, it is not clear to what extent other aspects of the policy to integrate chronic care have been implemented.^24^ As KI 2 explained, the actual application of the national policy depends on the political will and ability of the provincial departments and the district structures to comply. As a result, there is significant variation in implementation across the nine provinces. There has been some resistance to the change in approach, but this has diminished over time, and where the policy has been implemented, reports are that it has decreased waiting times and increased patient satisfaction. KI 2 reported that:

‘We’ve still got some people arguing that diabetes, HIV or mental health are too different … that the one is more important or complex to treat, but once you put them together and people do this for a while, it seems to work. We are getting less complaints now. So, we (the national department) are quite happy with the direction things are taking.’ (KI 2, Senior Official, National Department of Health)

## Theme 3: The current model for diabetes care in the Western Cape province

### Facility level

According to KI 1, who was an official in the Western Cape Department of Health with responsibility for policy implementation on chronic disease and geriatric care, the Department is working towards the implementation of the national policy to integrate chronic care. At the district level, the department has established multidisciplinary chronic disease management teams, which are led by family physicians and include medical officers, clinical nurse practitioners, pharmacists, social workers and rehabilitation staff. The other key informants confirmed that this team has the primary responsibility for diabetes care at the primary care clinic. In each larger geographical service area, there is an ‘area committee’, which is a large forum with representatives from all levels of the health-care service. Within those forums, there are different working groups, including one working on the integration of chronic disease care at a primary care level:

‘There has been lots of consultation. People are buying into this new policy direction. We have a new policy called the Integrated Management of Chronic Conditions (IMCC), which covers chronic communicable and non-communicable disease, as well as mental health …‘ (KI 1, Director, Western Cape Provincial Department of Health)

The policy commits to a whole of society approach to address social determinants and a systems approach to drive the reorientation of the health system to more effectively address NCDs. At the level of the health service, the following components are specified: the delivery of a comprehensive, integrated package of care using a life course approach; good clinical governance; and the provision of person-centred, self-management support. Central to achieving the goal of improved NCD outcomes is a productive interaction between a well-prepared and proactive provider and a well-informed, empowered patient.

### Diabetes Lifestyle Education Collaboration and Action programme

In line with this policy, the Diabetes Lifestyle Education Collaboration and Action (D-LECA) programme is being adapted to include other chronic conditions. Diabetes Lifestyle Education Collaboration and Action is currently being piloted in three community health centres in the metro district as a structured educational and self-management programme for newly diagnosed diabetic patients. According to the family physician interviewed, everyone in the multidisciplinary chronic care team plays their part in assisting diabetes patients with self-management. Planning is underway for a phased, scale-up of the adapted version of D-LECA to all facilities in the province. The intention is to combine the resources used in D-LECA and the HIV–ART programme to form a new, holistic and comprehensive self-management programme for chronic conditions. This would move away from a narrow focus on adherence, to empowering patients for self-care and lifestyle change: ‘D-LECA is about empowering people to get ready to self-manage. The bottom line is, we obviously want people to maintain good health’ (KI 1, Director, Western Cape Provincial Department of Health). A tender has been won by to the Department of Family Medicine and Primary care at the University of Stellenbosch to train a group of master trainers in patient-centred, brief behaviour change counselling. The plan is to upskill the HIV lay counsellors who already work in primary care facilities so that they can undertake education and counselling for a range of chronic conditions, principally diabetes, hypertension, COPD and HIV. Other categories of health workers will also receive this training.

### The chronic disease clubs

In response to the question regarding what diabetes self-management programmes or services are already in place in the province, all key informants mentioned the chronic disease clubs. While the stated aim of these clubs is to equip diabetic patients with the knowledge and skills to manage their diabetes, it was clear that currently, they focus mainly on adherence (as with the HIV clubs). The clubs can be either facility based or community-based and are geared to provide stable chronic disease patients with opportunities to access their medication and have their blood pressure and glucose monitored monthly. Some of them may offer limited health education. As with D-LECA, the Department’s intention is to broaden the scope of these clubs so that they offer support for the management of both communicable and non-communicable chronic disease. According to the family physician, these clubs are not working optimally at present:

‘They are not sufficiently effective. Whilst the nurses are respectful (towards the patients), they just want to get through the queue. Patients are also often keen to get home as soon as possible. Also, patients respect information from doctors more. She suggested that for the clubs to become more effective, ‘the nurses need ongoing training and help from lay workers. A specific doctor should also be allocated to a group and be used as a resource. The priority is to allow for sufficient time for staff to listen to patients – to their questions, experiences and concerns.’ (KI 3, Family Physician, Cape Town metro)

She added that the clubs should be for patients to share ideas on how to problem solve around common barriers, as well as for providing activities, such as cooking demonstrations, exercise classes, and fruit and vegetable co-operatives. She further suggested that positive role models and dieticians be invited periodically to give talks and distribute resources. The clinic manager thought it was essential that more staff received in-depth training in diabetes to be able to run these clubs more effectively as a means of promoting diabetes self-management. The health promoter’s view was that lay health workers should play a more prominent role in counselling in both the clubs and the clinics as:

‘[*T*]hey can help the healthcare providers get to know the challenges that these clients are facing. They consider the patient’s cultural background, their beliefs and values and help with language and cultural barriers. This makes the patients feel more comfortable.’ (KI 5, Health Promoter, Cape Town metro)

She added that she thought there should be greater community involvement in the running of the diabetes clubs or groups and that the Department should aim to establish clubs in every local community.

### Community-based services

At the community level, the provincial Departments of Health (DOH) have outsourced a package of services for chronic disease patients to NGOs. These NGOs are paid to render a service to a specific community. The NGO pays and manages its own staff, but the Department works closely with them and plays an oversight role. A community-based services (CBS) coordinator, who is a nurse employed by the DOH, liaises with the NGOs and social service sites run by the Department of Social Development in her area and reports to the CBS programme manager working in the DOH district management structure.

The package of services delivered by NGOs in the Western Cape province varies (as is the case in other provinces too):

‘It could be just providing medication, which has been pre-packaged by the Chronic Dispensing Units. On a monthly basis, patients will go to a group at a hall, a library or Service Site and fetch their medication. The NGO comes to fetch medication from the facility. There are tight control measures in place to ensure the medicines are kept under certain conditions and patients sign for their medication. The nurses oversee this process, and CHWs deliver the service. Or patients could also, in addition, receive education on healthy lifestyle and medication. These groups have different names: they might be called a Self-Management Group or an Empowerment Group or a Wellness Group.’ (KI 1, Director, Western Cape Provincial Department of Health)

However, again, the Department intends to expand the scope of the work done by the CHWs:

‘[*T*]he idea is for CHWs to do medication, as well as primary and secondary NCD prevention work in the community. CHWS currently working with HIV patients need to be upskilled to become general counsellors for chronic disease, just like the counsellors at the facility level. But now, we are not quite there.’ (KI 1, Director, Western Cape Provincial Department of Health)

**FIGURE 1:**
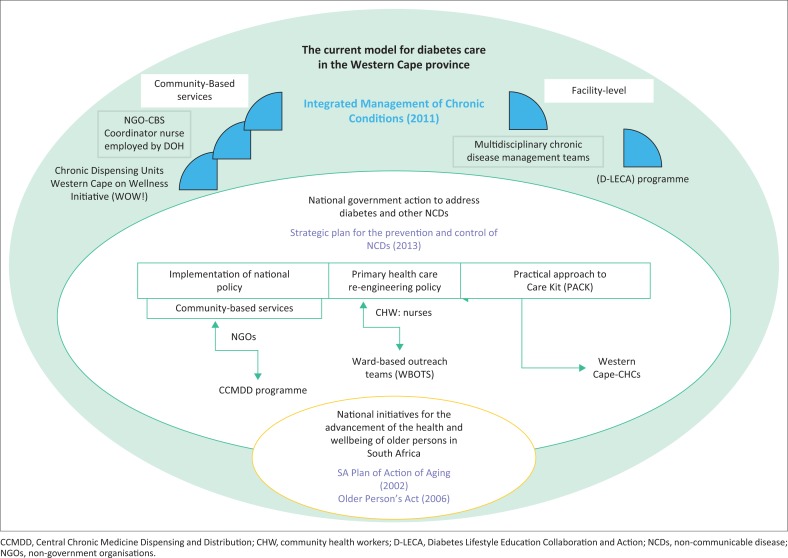
Main themes based on document review and key informant interviews.

The Western Cape Department of Health has also initiated the Western Cape on wellness initiative (WOW!) to prevent and reduce the risk of NCDs by promoting physical activity and a healthy diet in the community. Among its activities are training people to establish food gardens, convening a variety of popular physical activities in public spaces and organising health promotion media campaigns. Figure 1 summarises the main themes from the documentary review and the KI interviews.

## Theme 4: Self-management of diabetes among older patients

### Specific programmes for older patients

Within the primary health services in the Western Cape, there are no programmes that address the specific needs of older diabetes patients. KI 1 stated that:

‘[*O*]lder people are not addressed as a separate group. For me the only problem for older people is just the waiting times at the facility. We do have an approach that older people and people with disabilities must be taken out of the queue and helped, but it does not always happen like that. It is the responsibility of the staff at the facility to see whether they can reduce the waiting times for older people.’ (KI 1, Director, Western Cape Provincial Department of Health)

Fast-tracking older people in queues was confirmed as recommended practice by KI 2.

### Partnership of Departments of Health and Social Services

KI 1 also mentioned that the Department of Health partners with the Department of Social Development to run community-based groups for older people:

‘The Department of Social Development have groups for older people called Service Sites, where older people still living at home go to socialise and others which are run at old age homes. These are usually run by NGOs paid by Social Development. We have combined forces with them so that we can start providing medication and lifestyle education to chronic patients through NGOs at those sites. In that way, we can avoid starting another group. Social Development has also organised the Golden Games to help older people stay fit and active. There are heats throughout the year and then one big competition a year.’ (KI 1, Director, Western Cape Provincial Department of Health)

### Barriers to effective self-management for older persons

There was consensus among KIs that generally older patients wanted to be compliant, but they faced numerous barriers in managing their condition. ‘Many of them are hardly managing themselves: they are very dependent on their families in terms of preparing their meals, taking medication and attending follow up appointments’ (KI 5, Health Promoter, Cape Town metro). For clinicians, the most pressing issue was the lack of time for consultation with chronic patients, especially with those suffering from multi-morbidities. The family physician emphasised that currently, patients are given information in a didactic fashion with little consideration of their individual situation or daily lived experience:

‘Clinicians need to take time to listen to the patient. If the doctor does not understand the patient’s concerns or experiences, then the advice may be inappropriate. You also need time to go through the meds, so they understand the rationale and there needs to be shared decision-making so that there is agreement on what meds the patient is willing to take and what can be stopped.’ (KI 3, Family Physician, Cape Town metro)

To enhance her own capacity to manage older diabetes patients, she stressed that what she needed most was more time to develop a personal relationship with the patient and their family and to be able to offer a greater continuity of care. The health promoter argued that CHWs should be relied on to assist with self-management as they can spend more time getting to understand the patient’s context and capacity:

‘Patients would get more involved (in self-management) if they were in a partnership (with a health care provider), where they had time to ask questions and where their cultural backgrounds, values and beliefs were understood.’ (KI 5, Health Promoter, Cape Town metro)

**FIGURE 2:**
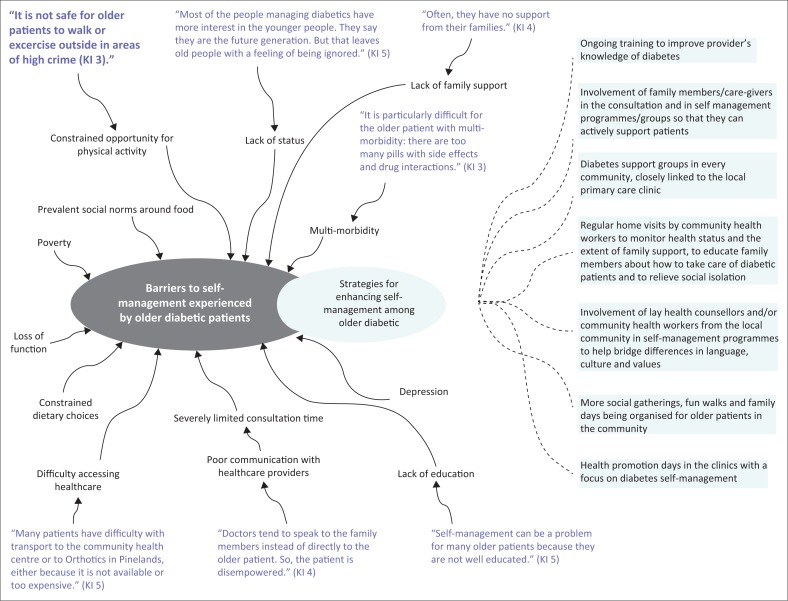
Barriers to effective self-management for older persons: The views of key informants.

He was worried that there was currently a shortage of staff to run self-management programmes and implored the Department to employ more diabetes educators. Further barriers to self-management commonly experienced by older patients are listed in Table 1-A2. The key informants also offered their ideas about how these barriers could be overcome (Figure 2).

## Discussion

The epidemiological transition in SA from acute, communicable disease to the prominence of chronic NCDs in an ageing population is resulting in an increased demand for long-term health care, institutional and family support.^23^ This situation is recognised by policy-makers, managers and practitioners alike, and there are multiple efforts both nationally and provincially to re-orientate the health-care system to focus more effectively on NCDs. This is evidenced by the re-engineering of primary care, which aims to provide integrated services for common chronic conditions, enhance the skills of health-care practitioners to manage chronic conditions and multi-morbidity, improve access to medicine through the CCMDD programme, strengthen self- management and form strong linkages with communities through NGOs and CHWs, to fill a critical gap in support services that are not provided by organised health care. It was clear from the key informant interviews that, while the re-organisation of primary care in this way may not be fully established, it is currently underway at least in the Western Cape province.

The policy-makers interviewed made it clear that it is highly unlikely that they would consider introducing a specific programme for older diabetes patients. However, this group does stand to benefit from many of the interventions being implemented as part of the NCD Strategic Plan. As multi-morbidity is a consistent feature of NCDs among the older individuals, the integration and coordination of chronic care services is indicated.^25^

The establishment of community-based services to provide self-management support, promote health and ease access to medicine, as well as providing home visits by district-based CHWs, helps overcome many of the barriers to care experienced by older patients. It enables services to be delivered early and close to home, decreases waiting times, reduces transport and other associated costs for individuals, and provides for continuity of care.^19^ For the health system, gains include cost saving with disease averted by health promotion and early detection, reduced facility visits and less overcrowding, making more time available for individual consultations.^19^ As one of our key informants stated, this would make it more feasible for practitioners to interact with patients and play a more active role in enhancing health literacy, health promotion and self-management. What was not mentioned by the key informants but which may be of equal importance is that practitioners gain appropriate communication skills and educational resources to effectively educate and counsel patients on lifestyle behaviour change and self-care. These factors have been reported by patients and health-care providers alike to be significant sources of frustration and to have a negative impact on the quality of NCD care.^26,27,28^

To cater for a growing population of older people, the health-care system also needs to be made more-age friendly, particularly at the primary care level. According to Samson, many health-care workers have negative attitudes towards older people, and as a result, manageable health issues are overlooked or attributed to the ageing process, resulting in low levels of functioning, poorer health outcomes and diminished quality of life. ^29^

The available research on the question of how to improve the care of older patients with diabetes highlights several important considerations. Poor health literacy related to low educational attainment and limited access to media among older patients is a known contributing factor to suboptimal diabetes outcomes among this group.^30^ Older patients may therefore require additional time for health education and self-management support, which is tailored to their cognitive and functional status (older patients with multiple co-morbidities are especially likely to become confused about their treatment). For example, a 2015 survey of diabetes patients > 60 years attending public sector primary health-care clinics in Cape Town found very poor knowledge of diabetes complications, its causes and self-management practices.^31^ This was both indicative of their low levels of formal education (67% had less than 5 years of schooling) and the extremely limited time and resources health-care providers had to educate and counsel diabetic patients.^32^ It has been argued that as older patients need more time for communication with providers, health services should be more responsive to and centred on their needs and that support needs to be provided in the community from health-care workers who understand the local context and language.^33,34^ Community-based support groups run by lay health workers have been shown to be particularly helpful in providing such extended support for older people. For example, Gilden et al.^35^ reported that older diabetic patients who attended a series of educational and social support groups had better knowledge, greater family involvement and improved quality of life; experienced less depression and stress and achieved greater glycaemic control than a control group. It is also recommended that there be greater recognition of and support for the important role of the family and non-professional caregivers in keeping older patients functionally independent and at home, thereby reducing health and social care costs.^36^ They should be included in support groups and consultations with health-care providers, where the older patient’s ability to self-manage is frequently reviewed.^33^ Older patients have been found to be at high risk of nutritional deficiency, with under-nutrition and over-nutrition, as well as food insecurity.^37,38,39^ This suggests that nutritional status and the risk of hypoglycaemia should be included in such reviews.^40^ As a group, older diabetic patients are also at a higher risk of untreated depression and/or anxiety, indicating that greater attention should be afforded to the screening and treatment of mental health among older patients with diabetes.^35^

## Conclusion

While there have been some significant policy interventions pertaining to the protection of the health and welfare of older persons in SA, the needs of this vulnerable group remain relatively low on the list of priorities in terms of focus and resource allocation. In this context, older people, as a distinct group, are also not a strong focus in current health policy relating to the provision of NCD care. However, the various initiatives currently underway to re-engineer the health-care system in SA to more effectively deal with NCDs, will go some way to meeting the identified needs of older diabetic patients and to addressing their barriers to care. However, as part of this re-modelling exercise, it is perhaps opportune for the health department to consult older chronic care patients and involve them in decision-making and the planning of services. This article alerts policy-makers and clinicians to some of the specific issues considered to be pertinent and important in the care and management of older diabetic patients. Many of these would also be applicable to older patients with other chronic conditions.

## Appendix 1

**TABLE 1-A1:** Sources for document review.

Title	Author and date of publication	Source
White Paper for Social Welfare	Ministry of Social Development 1994	www.gov.za/sites/default/files/White_Paper_on_Social_Welfare_0.pdf
*Older Person’s Act* (Act 13 of 2006)	SA Parliament	www.westerncape.gov.za/legislation/older-persons-act-13-2006
SA National Plan of Action on Ageing	Ministry of Social Development 2002	www.tafta.org.za/images/SAPlanofActiononAgeing.pdf
South Africa’s Progress on the Implementation of the Madrid Plan of Action on Ageing	Ministry of Social Development 2007	http://libguides.lib.uct.ac.za/c.php?g=214526&p=1579027
Policy implications and challenges of populations ageing in South Africa	Goodrick WF, 2013	scholar.google.com/scholar
Vulnerable Groups Series II: The Social Profile of Older Persons, 2011–2015	Statistics South Africa 2017	http://www.statssa.gov.za/publications/Report%2003-19-03/Report%2003-19-032015.pdf
Society for Endocrinology, Metabolism and Diabetes of South Africa (SEMDSA) issued the first edition of the Consensus Guidelines for the management of type 2 diabetes	JEMDSA, 2017, Vol 22, no 1, Supp 1, pages S1-S196	www.semdsa.org.za/images/647-4385-1-pb.pdf
Master’s Thesis in Sociology, University of the Free State, 2013: Policy implications and challenges of population ageing in South Africa	Wade Francis Goodrick	http://scholar.ufs.ac.za:8080/xmlui/handle/11660/1879
The Samson Institute for Ageing Research (SIFAR)	-	http://www.sifar.org.za
SA Strategic Plan for the Prevention and Control of NCDs	-	http://www.hsrc.ac.za/uploads/pageContent/%20proof.pdf
SA Health Review 20th edition, 2017	2017	http://www.hst.org.za/publications/South%2%20Version.pdf

SA, South Africa; NCDs, non-communicable diseases.

## Appendix 2

**TABLE 1-A2:** Barriers to self-management experienced by older diabetic patients.

Barriers	Patient experience
Side effects of medication	‘Many patients experience side effects with Metformin. We get tons of returned meds in the pharmacy bins. Patients want to be seen as “good patients” and just accept medication, even if they don’t intend using them’. (KI 1)
Loss of function	‘With poor vision and motor control it is difficult for them to give themselves injections and examine their own feet’. (KI 1)
	‘Many patients have cognitive decline that isn’t diagnosed or acknowledged, so they don’t understand clearly or get confused’. (KI 1)
	They get forgetful and can’t remember which pills are for what and if they have even taken them’. (KI 1)
Constrained dietary choices	‘The elderly have fewer choices because they are often living with their children and have no say in the shopping or cooking. They just eat what there is to avoid feeling like a burden’. (KI 5)
	‘They think they need to eat special foods labelled ‘Diabetic’ and these are expensive. Fruit and vegetables are also expensive if you don’t have transport to Wholesalers like Fruit&Veg’. (KI 5)
	‘High carb foods are relatively cheap for the satiety they give. For example, if one is hungry a loaf of bread and polony is cheap and filling’. (KI 5)
Prevalent social norms around food	‘Rejecting food that is offered is seen as rude, especially at a social function like a wedding or birthday. People do not usually cater for diabetics: big life events are celebrated with calorie dense, sugary food’. (KI 5)
Poverty	‘Many of my older patients wear cheap shoes that squeeze their toes and give poor support because they cannot afford sports shoes with thick soles and a wide toe box’. (KI 3)
	‘It is hard to prioritise good diabetic control, when the emphasis is on just living day to day’. (KI 3)
Lack of education	‘Self-management can be a problem for many older patients because they are not well educated’. (KI 4)
Lack of family support	‘Often, they have no support from their families’. (KI 3)
Constrained opportunity for physical activity	‘It is not safe for older patients to walk or exercise outside in areas of high crime’. (KI 2)
Difficulty accessing health care	‘Many patients have difficulty with transport to the community health centre or to Orthotics in Pinelands, either because it is not available or too expensive’. (KI 2)
	‘Some of them are not so mobile and cannot get to the healthcare facility, so they need special care to accommodate them’. (KI 5)
Severely limited consultation time	‘Older patients need longer consultations but when you are seeing 35 patients a day, there is no time’. (KI 3)
	‘It is very hard to explain a complex condition with complex therapy in the time allotted and doctors often don’t communicate well’. (KI 3)
Poor communication with health-care providers	‘Generally, communication between providers and patients is very poor. Staff are sometimes impatient and disrespectful, because they are burnt out’. (KI 2)
	‘Providers tend to use medical terms which patients are not acquainted with. Patients would prefer simple explanations’.
	‘The staff should come down to the patient’s level so that they can feel they are part of the discussion about their health’. (KI 5)
	‘Doctors tend to speak to the family members instead of directly to the older patient. So, the patient is disempowered’. (KI 3)
Lack of status	‘Patients with chronic disease are undervalued by the health system’. (KI 2)
	‘Most of the people managing diabetics have more interest in the younger people. They say they are the future generation. But that leaves old people with a feeling of being ignored’. (KI 5)
	‘I am interested in managing diabetes in older people as I think they have real problems in managing themselves. Proper education should be provided to them to avoid them feeling rejected due to old age’. (KI 5)
Depression	‘Patients are often depressed, and this affects their motivation to live healthily’. (KI 3)
Multi-morbidity	‘It is particularly difficult for the older patient with multi-morbidity: there are too many pills with side effects and drug interactions’. (KI 3)
	‘Older patients are likely to be taking more than one chronic medication, which means they need closer monitoring as there may be drug interactions’. (KI 5)
	‘Each co-morbidity affects the other. For example, a patient with Osteoarthritis or Chronic Obstructive Pulmonary Disease finds it difficult to exercise’. (KI 3)
	‘Food that might be good for one condition, may not be good for another. For example, diabetic patients should eat more greens, but a patient on warfarin should avoid spinach because it has vitamin K, which thickens the blood’. (KI 5)
	‘Once you have checked on all the different conditions, there is little time to focus on diabetes. Patients with co-morbidities especially need longer consultation times’. (KI1)
